# Ecosystem multifunctionality, maximum height, and biodiversity of shrub communities affected by precipitation fluctuations in Northwest China

**DOI:** 10.3389/fpls.2023.1259858

**Published:** 2023-09-25

**Authors:** Lan Du, Shengchuan Tian, Jing Sun, Bin Zhang, Xiao-Han Mu, Lisong Tang, Xinjun Zheng, Yan Li

**Affiliations:** ^1^ State Key Laboratory of Desert and Oasis Ecology, Xinjiang Institute of Ecology and Geography, Chinese Academy of Sciences, Urumqi, Xinjiang, China; ^2^ University of Chinese Academy of Sciences, Beijing, China; ^3^ Fukang Station of Desert Ecology, Chinese Academy of Sciences, Fukang, Xinjiang, China; ^4^ State Key Laboratory of Subtropical Silviculture, Zhejiang A and F University, Hangzhou, Zhejiang, China

**Keywords:** ecosystem multifunctionality, maximum height, shrub communities, dryland, biodiversity

## Abstract

**Introduction:**

Dryland ecosystems face serious threats from climate change. Establishing the spatial pattern of ecosystem multifunctionality, maximum height and the correlation of biodiversity patterns with climate change is important for understanding changes in complex ecosystem processes. However, the understanding of their relationships across large spatial areas remains limited in drylands.

**Methods:**

Accordingly, this study examined the spatial patterns of ecosystem multifunctionality, maximum height and considered a set of potential environmental drivers by investigating natural shrub communities in Northwest China.

**Results:**

We found that the ecosystem multifunctionality (EMF) and maximum height of shrub communities were both affected by longitude, which was positively correlated with the precipitation gradient. Specifically, the EMF was driven by high precipitation seasonality, and the maximum height was driven by high precipitation stability during the growing season. Among the multiple biodiversity predictors, species beta diversity (SD-beta) is the most common in determining EMF, although this relationship is weak.

**Discussion:**

Unlike tree life form, we did not observe biodiversity-maximum height relationships in shrub communities. Based on these results, we suggest that more attention should be paid to the climatical fluctuations mediated biodiversity mechanisms, which are tightly correlated with ecosystem’s service capacity and resistance capacity under a rapid climate change scenario in the future.

## 1 Introduction

While providing multiple ecosystem servises, ecosystems are also under pressure of environmental harshness. The ability of ecosystems to maintain functioning and resist environment harshness, known as ecosystem multifunctionality (EMF) and resistance capacity, respectively (Maestre et al., 2012; Marks et al., 2016; Gross et al., 2017). Since the 1990s, positive correlations between biodiversity and ecosystem nultifunctionality have been reported in a number of biodiversity manipulation experiments (Byrnes et al., 2014; Gross et al., 2017). To date, a body of researches have found that this relationships may be weak (Ratcliffe et al., 2017), neutral (Grace et al., 2007), and hump-shaped (Fei et al., 2018). This diverse linkages between biodiversity and ecosystem multifunctionality may be influenced by altered biological and abiotic conditions, which are greatly restricted by geographical constraints (Symstad et al., 2003). The persistence of tree life form to environmental harshness reflected at multidimensional space, such as the axes of seed mass, leaf life span, tree height, wood density and so on (Westoby et al., 2002). In functional ecology, it is a common approach to characterize the responses of individuals to environmental harshness through physiological or morphological characteristics that are operable and easily measured. The maximum height, as an integrated reflection of tree life form to resources availability, has been extensively used to evaluate the adaptability of plants to environmental harshness (Givnish et al., 2014; Stahl et al., 2014). Recently, Marks et al. (2016); Marks et al. (2017) showed that tree diversity was positively correlated with maximum tree height on both alpha and beta scales, and these linkages covaried with the environment harshness. However, these linkages between ecosystem multifunctionality, plant resistance capacity, and biodiversity were proposed in the study of trees. Do these conclusions also apply to shrubs? Comparing with tree life form, shrubs tend to suffer from long-term constraints by environmental harshness, but we seem to pay insufficient attention to them. For example, in Northwest China, water scarcity is a constant threat to plant survival, and the higher the longitude, the faster and more intense the changes in water supply of shrub communities (Zhang S. et al., 2022; Guo et al., 2023). There remains a lack of knowledge regarding how the ecosystem multifunctionality, maximum height, and biodiversity of shrub communities vary with this spatial gradient. Filling this knowledge gap could provide important insights into the response of most ecological processes in arid regions to climate change (Zhou et al., 2019; Yu et al., 2021; Norman et al., 2022).

At different attribute levels, biodiversity can be divided into species diversity, functional diversity, which conveys ecosystem functions, and phylogenetic diversity, which reflects evolutionary history (Richter et al., 2021). Previous studies have mainly been conducted at the alpha scale and have found that the impact of alpha diversity on ecosystems is mainly achieved through selection and complementary effects (Garcia-Palacios et al., 2018). Recently, with increasing biotic homogenization, beta-scale diversity has attracted widespread attention. Unlike alpha diversity, high beta diversity can promote ecosystem multifunctionality through the different contribution of local species caused by trait variation (Grman et al., 2018). For example, species beta diversity (SD-beta) has been used to classify the floristic regions of Baja California (Garcillan and Ezcurra, 2003). Some studies have suggested that functional diversity may be more effective than species diversity in predicting ecosystem functions (Cadotte, 2017; Laughlin et al., 2020; Guo et al., 2023), perhaps because certain functional characteristics are related to how species participate in competition and use resources (Valencia et al., 2015). Similarly, phylogenetic diversity (PD-beta) is an effective indicator of phylogenetic distance at the species and community levels. As such, it allows us to link individual processes (i.e., environmental filtering and biological interactions) to more regional dynamics (i.e., dispersal and trait evolution) (Qian et al., 2021). Although many studies have shown that alpha and beta diversities are critical for maintaining ecosystem functioning, little is known about their relative importance and synergy in multiple ecosystem functions.

Recently, with the changing global environment, the interactions between ecosystem processes and environmental drivers have attracted significant attention (Hautier et al., 2015; White et al., 2022; Qiao et al., 2023). Specific environmental drivers may cause long-term impacts on ecosystem functions and sustainability, and exhibit greater spatial heterogeneity with geographic gradients (Loarie et al., 2009; Nishizawa et al., 2022). This study identified three environmental factors affecting the relationships between ecosystem multifunctionality, maximum height, and biodiversity of shrub communities: (i) Resource conditions (i.e., precipitation and temperature), which represent the overall resources obtained from a habitat to maintain plant growth and have been proven to be critical in stabilizing ecosystem functioning in arid regions (Zhang X. et al., 2022; Guo et al., 2023). (ii) Climate stability during the growing season is critical for maintaining biodiversity and ecosystem functioning in drylands (Souza et al., 2016; Lu et al., 2019). Studies have found that climate stability could affect community functioning by reducing both SD-alpha and SD-beta in temperate grasslands (Zhang et al., 2019), and another study has shown that climate stability is driven by longitude in forest ecosystems (Qiao et al., 2023). (iii) Climate seasonality is believed to be an underestimated factor affecting ecosystem functioning at the macro scales (Liang et al., 2022; Du et al., 2023). Seasonal climates may cause large disturbances to community functioning by influencing the growth and survival of plant (Escobedo-Kenefic et al., 2020; Liang et al., 2022). However, few studies have comprehensively considered the effects of these environmental factors on ecosystem processes, which may bias our understanding of their stabilizing effects on ecosystem functions on a broader spatial scale.

To fill this knowledge gap, we conducted transect observations of shrub communities in Northwest China. This sampling enabled us to understand the spatial dynamics of ecosystem multifunctionality and maximum height in shrub communities, particularly their linkages with multi-scale biodiversity. We expect to answer the two questions: (i) Is precipitation fluctuation the main driving force for interpreting spatial gradients and, ultimately, ecosystem multifunctionality and maximum height in shrub communities? (ii) How does the climatic environment constrain linkages between ecosystem multifunctionality, maximum height, and biodiversity in shrub communities? We expect this study to strengthen our understanding of spatial changes in ecosystem processes in shrub communities, thereby providing insights into the landscape management.

## 2 Materials and methods

### 2.1 Study sites and vegetation inventory data

**Figure 1:**
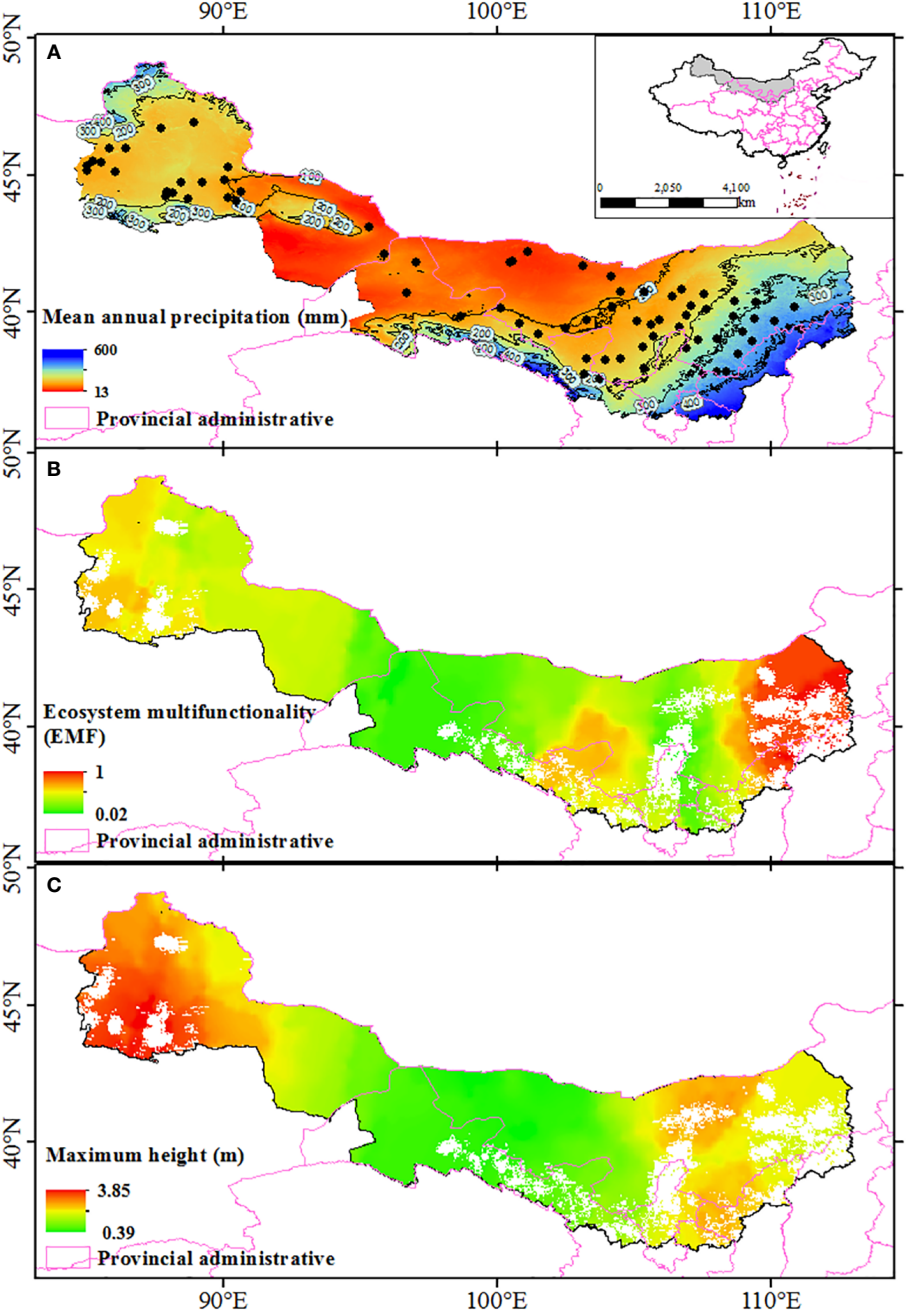
Geographic distribution of the sampling sites along the precipitation gradient in Northwest China **(A)**, the geographical patterns of ecosystem multifunctionality **(B)**, and maximum height along sampling area **(C)**. Blank areas in **(B, C)** represent densely populated cities and are not considered as natural ecosystems. Spatial interpolation is accomplished by Kriging interpolation. The map are edited based on standard national boundary (GS(2016)1600).

Study was conducted along a west-east transect (84°58’ E ~ 111°6’ E, 37°26’ N ~ 46°55’ N) spanning a broad range of dryland ecosystems in Northwest China (**Figure 1A**). The Ulanbuhe, Kubuchi, Mawusu, Tengri, Gurbantungut, and Badangilin deserts were included in this study. In this region, mean annual precipitation ranges from 38 to 403 mm, mean annual temperature ranges from 3.95 to 9.86 °C, and the species alpha diversity ranges from 2 to 11 (average value is 4). More than 78.9% of the rainfall occurs during the growing season, which directly affects vegetation survival in drylands. This region is affected by both westerlies and monsoon climates (**Figure 1A**). The western part of this region is affected by the westerly airflow from the Atlantic Ocean, which is blocked by the eastern Tianshan Mountains and cannot continue eastward. However, the eastern region is influenced by the prevailing summer monsoon, and moist air from the Pacific Ocean replenishes this region with precipitation.

Field sampling was carried out during the growing season (June to September) in 2021. A total of 33 shrub species from 77 natural shrub communities were sampled (**Table S1**). To minimize human disturbance, sampling points near the nature reserves and ecological monitoring stations were chosen. Four 20 m×20 m subplots with intervals of no less than 1 km were randomly established in each sampling plot. All living plant individuals in each subplot were recorded, separately. The plant height (H) and crown diameter (CD) of all shrub plants in the subplots were also investigated. The coordinates (longitude, latitude, and altitude) of each site were recorded using a geographical positioning system (Thales, USA). At the end of the investigation, plant branchlets in the subplot were sampled (with no fewer than four replicates for each species) and stored in a crisper with ice cubes. After being transported to the hotel, we divided plant samples into two parts. One part was used to scan the leaf area (LA), leaf length (LL), and leaf width (LW). I_MAGE_J (1.8.0v for PC; W. Rasband, National Institute of Health, Bethesda, MD, USA) was used for image processing. Finally, the scanned leaves were oven-dried at 60°C to obtain leaf dry mass (LDM). Specific leaf area (SLA) was calculated using the formula SLA=LA/LDM. The other part was deoxidized at 105 °C for 30 min and oven-dried at 80 °C for 12 h and their chemical characteristics determined. Three surface soil (0–20 cm) samples at each plot were also collected and mixed them as composite sample. After air-dried, the soil samples were removed plant litter and gravel by pass a 2 mm sieve.

The dried plant samples were ground using a Ball Mill (NM200 Ball Mill; Retsch, Haan, Germany) and the air-dried soil samples were analyzed. Organic carbon was determined by an external heating method with potassium dichromate, total nitrogen was determined by the Kjeldahl method, and total phosphorus was determined by the HClO_4_-H_2_SO_4_ ammonium molybdate-ascorbic acid method.

### 2.2 Multiple and scales of biodiversity

In this study, species richness was used to represent species alpha diversity, because species richness is the most intuitive measure of species diversity (Valencia et al., 2015). Functional dispersion (FDis) was used to represent functional alpha diversity (Lavorel et al., 2008). We calculated FDis using LW, LL, SLA, H, and CD as functional traits, all of which are critical for characterizing the assimilation and tolerance abilities of plants (Gross et al., 2017). Phylogenetic tree was constructed using the list of investigated plants, and the mean nearest taxon distance (MNTD) was used to express phylogenetic alpha diversity (Jin and Qian, 2022). Beta-scale diversity refers to the differences in community composition, which are also reflected in species, function, and phylogeny (Grman et al., 2018). To calculate the species beta diversity, the Bray-Curtis similarity index was used to represent the compositional differences in each community (Blomberg et al., 2003). Functional beta diversity was characterized by the Euclidean distance between the five plant traits in different communities (Yan et al., 2020). Finally, phylogenetic beta diversity was indicated by pairwise distances between pairs of loci based on the phylogenetic trees for all sites.

### 2.3 Ecosystem multifunctionality and plant resistance capacity

Ecosystem multifunctionality describes the functioning of ecosystem within a certain time and space (Valencia et al., 2015). Six functional traits divided into three functional categories (C cycling, N cycling, and P cycling) were used for this calculation. In this study, we adopted the approach of Maestre et al. (2012) to calculate ecosystem multifunctionality, and dues to its analytical robustness, it has been widely used by ecologists (Byrnes et al., 2014; Hu et al., 2021). To apply this methodology, all sites were normalized (Min-Max normalization) for the predictor variables and weighted according to each subplot. Second, normalized variables were averaged to obtain the ecosystem multifunctionality.

Plant resistance capacity reflects the ability of plant to adapt from benign to harsh environmental conditions at regional scales (Marks et al., 2016). In this study, we used the maximum height measured in each plot (four subplots) to represent plant resistance capacity in this region. This is because the integrated maximum tree height can comprehensively reflect the resistance and resilience of the ecosystem as well as the status of the structure and function of the ecosystem fluctuating within a certain threshold range over time (Gross et al., 2017; Ouyang et al., 2023). In addition, maximum height is related to the multidimensional space of plant light capture, water conduction, and tolerance to harsh environment, which has been confirmed in many studies (Poorter et al., 2012; Marks et al., 2016; Prado-Junior et al., 2017).

### 2.4 Climatic data

The climate-related environmental information can be classified into three categories: resource conditions, climatic stability, and climatic seasonality. Resource conditions were calculated based on the effects of heat and water on plant growth (Ma et al., 2017; Qiao et al., 2023). In this study, the mean annual precipitation and mean annual temperature were obtained from the WorldClim2 dataset with a resolution of 1× 1 km for the years of 1990-2017 (Fick and Hijmans, 2017), and used to represent the heat and water resources of each plot, respectively. Climate stability is characterized by the inter-annual stability of precipitation and temperature during the growing season (Zhang et al., 2018). The stabilities of temperature and precipitation were calculated by inverting the inter-annual variation coefficients of monthly mean temperature and monthly total precipitation during the growing season (May, June, July, August, and September) from 1990 to 2017, respectively. Climate seasonality is represented by intra-annual precipitation seasonality and temperature seasonality. The precipitation and temperature seasonality were derived from a bioclimatic variable dataset (1 km × 1km) (Fick and Hijmans, 2017). Climate seasonality was expressed as intra-annual variation coefficients of 12 months from 1990 to 2017. All climatic data were extracted using A_RC_GIS v.10.7 (ESRI, Redlands, CA, USA).

### 2.5 Statistical analysis

All statistical analyses were conducted in R 4.1.3 (R Core Team, 2021). To meet the normality requirements of data analysis, we performed natural log_e_ transformations of all explanatory variables. First, we used linear regression analysis (LRA) to examine how geographical gradients affect ecosystem multifunctionality and maximum height at a regional scale. If the relationship was significant, partial LRA was performed by extracting the residuals and testing the relationship between the residuals and each individual predictor variable.We also used LRA to explain how geographical gradients affect climate and multi-scale biodiversity. Finally, LRA was used to test the interference between the pairwise climate predictors (i.e., temperature seasonality and precipitation seasonality). If the interference was significant, partial LRA was performed by extracting the residuals and testing the relationship between the residuals and each individual predictor variable.

To reveal the effects of various environmental factors on ecosystem multifunctionality and maximum height, we selected the resource conditions (mean annual precipitation and mean annual temperature), climate stability (temperature stability and precipitation stability), climate seasonality (temperature seasonality and precipitation seasonality), three biodiversity variables (SD-beta, FD-beta, and PD-beta), and two geographic variables (latitude and longitude) from the initial liner regression model. First, to obtain the relative influence of each predictive variable through the standardized regression coefficient, predictor variables were standardized (average=0 and standard deviation [SD]=1) before conducting a multiple linear analysis. To avoid multicollinearity, predictors with more than five variance inflation factors (VIF>5) were excluded by using vif function in R package Car (De Souza et al., 2019). All possible combinations of predictors were calculated. Models were ranked according to their corrected AIC, AICc. In the case of several comparable models, the average of all models with ΔAICc (calculated from the model with lowest AICc)<2 was selected by using model average function in R package MuMIn (Burnham and Anderson, 2002). The relative importance of the predictors was grouped into five identifiable variance fractions: geography, biodiversity, resource conditions, climatic stability, and climatic seasonality (Yuan et al., 2021).

To illustrate the hypothesized associations, we developed a piecewise structural equation model (pSEM) framework based on prior knowledge (**Figure S1**). First, to reduce the model complexity, variables representing resource conditions, climatic stability, and climate seasonality were summarized by running principal component analysis. Then we selected the first component (PC1, with the explanation of total variation ranges from 58.6% to 92.05%) to reduce the complexity in the pSEM. When the p-values of Fisher’s C were greater than 0.05, the fit was considered reasonable (Shipley, 2009; Lefcheck, 2016). In this section, to obtain the standardized path coefficient, we standardize (average=0 and SD=1) all explanatory variables in the pSEM. The pSEM was established by using R package piecewiseSEM (Lefcheck, 2016).

## 3 Results

### 3.1 Longitudinal gradients of ecosystem multifunctionality, maximum height, climate, and biodiversity

**Figure 2:**
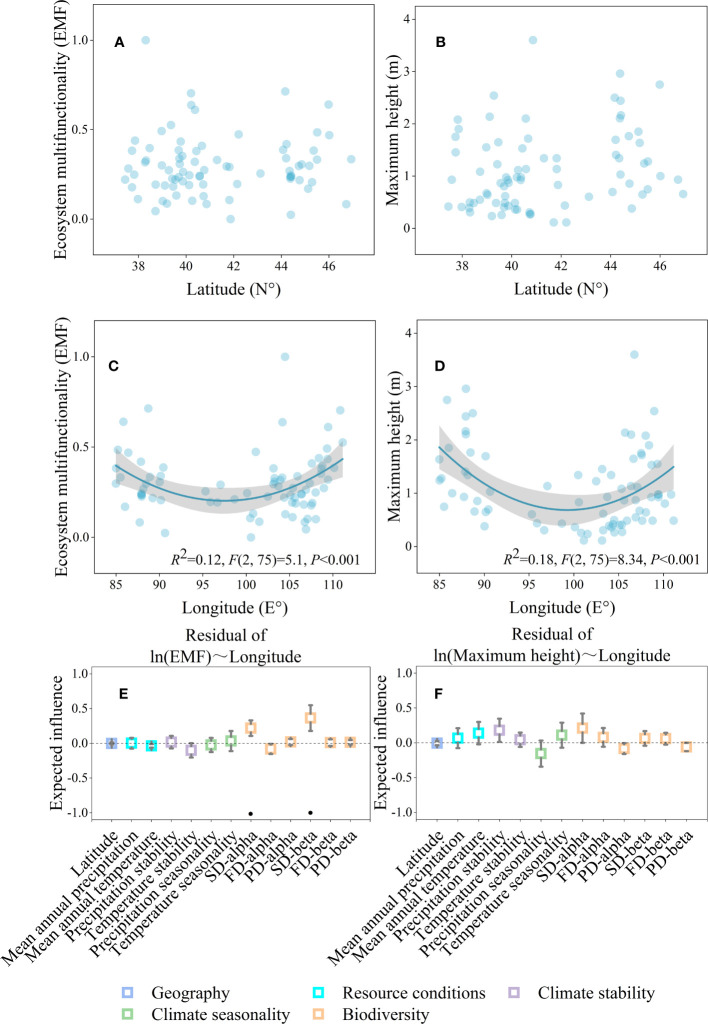
Geographical gradients affecting ecosystem multifunctionality and maximum height. Relationships between geography gradients and ecosystem multifunctionality **(A, C)** and maximum height **(B, D)**; the partial LRA of ecosystem multifunctionality **(E)** and maximum height **(F)**, and each independent variable relationship after controlling the effect of longitude. Shaded areas in **(C, D)** represent 95% confidence intervals. Squares and error bars in **(E, F)** represent the estimated means and 95% confidence intervals of liner regressions, respectively. In **(E, F)**, significance level was expressed as ·*P* < 0.1.

**Figure 3:**
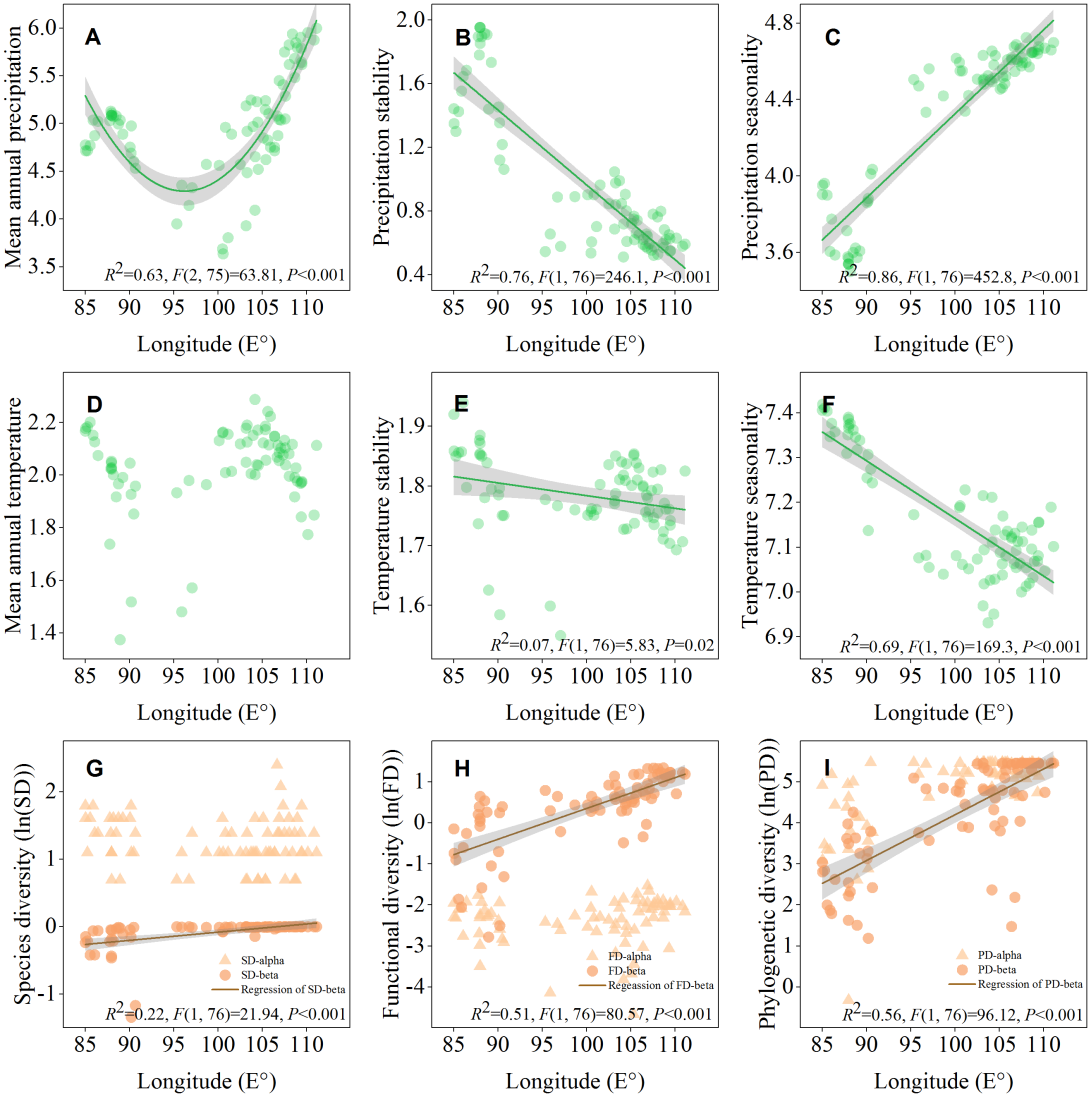
Longitudinal patterns of environmental variables **(A–F)** and biodiversity **(G–I)** in Northwest China. Shaded areas represent 95% confidence intervals.

The geographical distribution map showed that ecosystem multifunctionality and maximum height was positively correlated with the mean annual precipitation, and all of them affected by longitude (**Figure 1**). The results of the LRA also indicated that both the ecosystem multifunctionality and maximum height were nonlinearly (from negative to positive) correlated with longitude (**Figures 2C, D**, *P*<0.05), but not latitude (**Figures 2A, B**, *P*>0.05). The ecosystem multifunctionality was only positively correlated with SD-alpha and SD-beta after controlling the effect of longitude (**Figure 2E**, *P*<0.1). However, maximum tree height was not significantly associated with any of the predictor (**Figure 2F**, *P*>0.1). Many climatic variables were tightly correlated with longitude (**Figure 3**). Specifically, precipitation stability, temperature stability, and temperature seasonality decreased with increasing longitude (**Figures 3B, E, F**). Mean annual precipitation (nonlinear) and precipitation seasonality increased with longitude (**Figures 3A, C**). In terms of biodiversity, only SD-beta, FD-beta, and PD-beta increased with increasing longitude (**Figures 3G–I**).

### 3.2 Precipitation fluctuations in determining biodiversity, ecosystem multifunctionality, and maximum height

**Figure 4:**
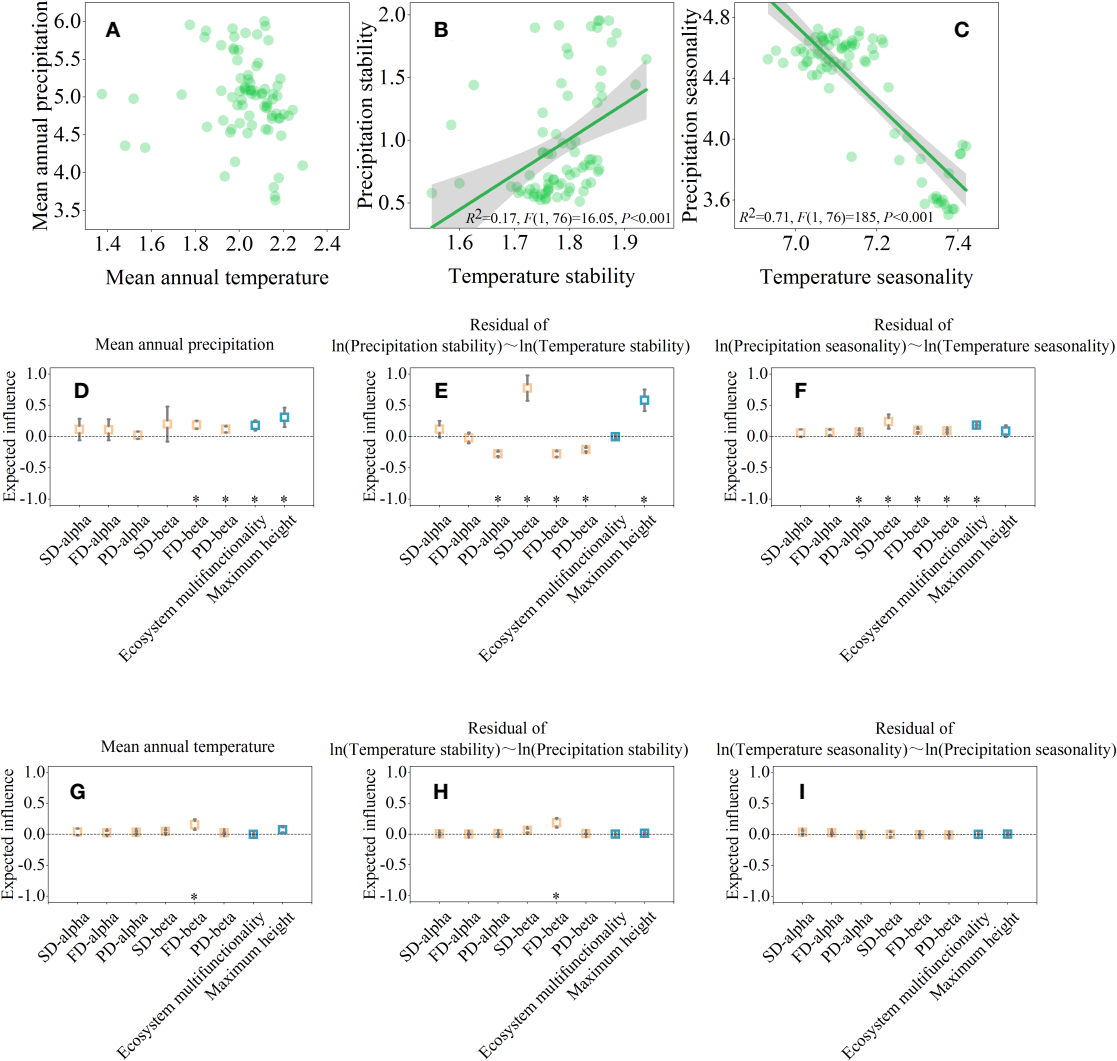
Climate drives ecosystem multifunctionality, maximum height, and biodiversity across spatial scales. The pairwise relationship between climate drivers **(A–C)**. And their partial linear relationships with ecosystem multifunctionality, maximum height, and biodiversity after controlling the effect of x-axis **(D–I)**. In **(D–I)**, significance level was expressed as *P < 0.05.

The LRA results showed that interference between precipitation stability and temperature stability, precipitation seasonality and temperature seasonality were significant, except for mean annual precipitation and mean annual temperature. (**Figures 4A–C**, *P<*0.05). Mean annual precipitation was positively correlated with FD-beta, PD-beta, ecosystem multifunctionality, and maximum height (**Figure 4D**). Whereas mean annual temperature was only positively correlated with FD-beta (**Figure 4G**). Partial LRA revealed that, after controlling the effect of temperature stability, precipitation stability was negatively correlated with PD-alpha, FD-beta, and PD-beta, but positively correlated with SD-beta and maximum height (**Figure 4E**). In addition to precipitation stability, many traits are also affected by precipitation seasonality. For example, PD-alpha, SD-beta, FD-beta, PD-beta, and ecosystem multifunctionality were positively correlated with precipitation seasonality, after controlling the effect of temperature seasonality (**Figure 4F**). However, only a few temperature-driven relationships (i.e., mean annual temperature, temperature stability, and temperature seasonality) were observed (**Figures 4G–I**).

### 3.3 Different driving forces between ecosystem multifunctionality and maximum height

**Figure 5:**
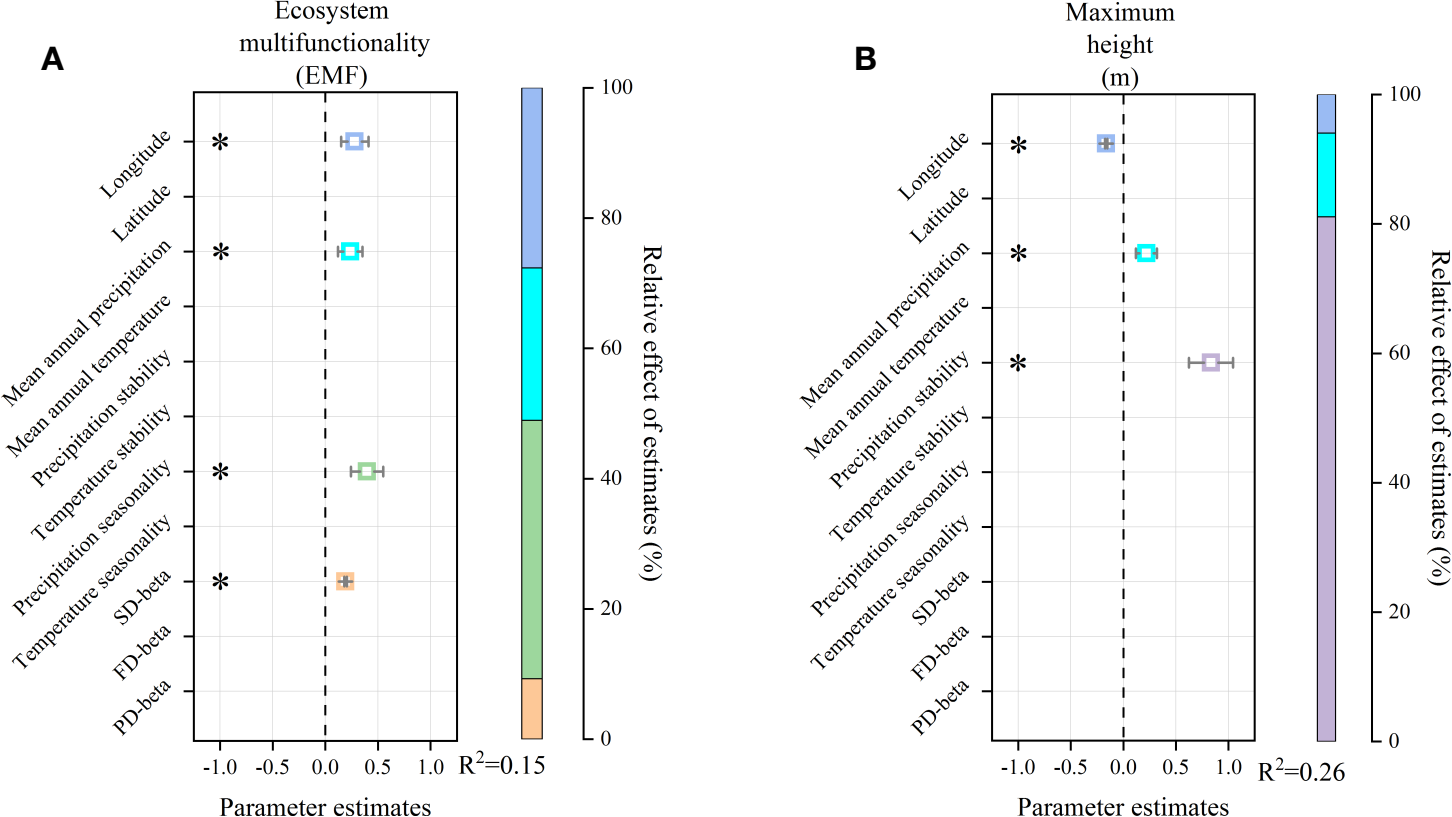
Multiple liner regression models of ecosystem multifunctionality and maximum height. In **(A)**, on the left, squares and error bars represent the standardized regression coefficients and 95% confidence intervals of model predictors, respectively. Significance level was expressed as *P < 0.05. On the right, the variations explained by different variable types and the adjusted *R* ^2^ of the models are shown. In **(B)**, the same as in **(A)**.

Multiple LRA showed that ecosystem multifunctionality was positively affected by longitude, mean annual precipitation, precipitation seasonality, and SD-beta (**Figure 5A**). Geography (longitude), resource conditions (mean annual precipitation), climate seasonality (precipitation seasonality), and biodiversity (SD-beta) explained the 27.67%, 23.42%, 39.65%, and 9.27% variation, respectively (**Figure 5A**, *R* ^2 ^= 0.15). The maximum height was negatively affected by longitude, but positively correlated with mean annual precipitation and precipitation stability (**Figure 5B**). Geography (longitude), resource conditions (mean annual precipitation), and climate stability (precipitation stability) explained the 5.98%, 12.9%, and 81.11% variation, respectively (**Figure 5B**, *R* ^2 ^= 0.26).

**Figure 6:**
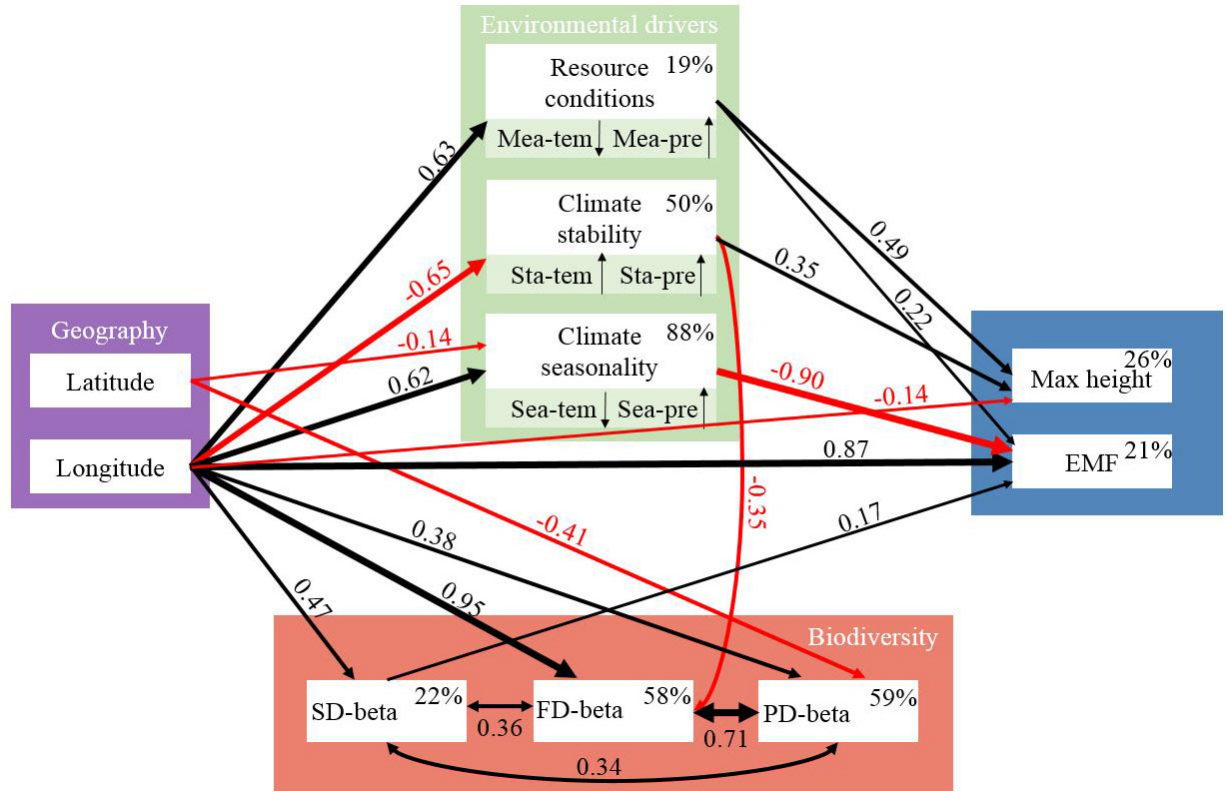
Final pSEM exploring potential ecological drivers of ecosystem multifunctionality and maximum height across scales (*Fisher’s C*=37.47, *df*=44, *p*=0.746, *AIC*=107.47). Single-headed arrows and double-headed arrows represent causal pathways and co-varying relationships, respectively. Black and red lines represent positive and negative correlations (*P* < 0.05), respectively. Thickness of the arrows and the standardized path coefficients indicate the magnitude of path relationships. Percentages next to the endogenous variables represent the *R* ^2^. ‘↑’ and ‘↓’ represent the positive and negative correlations between climate predictors and the corresponding PC1, respectively. Mea-pre, mean annual precipitation; Mea-tem, mean annual temperature; Sta-pre, precipitation stability; Sta-tem, temperature stability; Sea-pre, precipitation seasonality; Sea-tem, temperature seasonality; SD-beta, species beta diversity; FD-beta, functional beta diversity; PD-beta, phylogenetic beta diversity; EMF, ecosystem multifunctionality; Max height, maximum height.

The results of pSEM revealed that longitudinally affected environmental gradients significantly influenced both ecosystem multifunctionality and maximum height, but biodiversity was found to be only correlated with ecosystem multifunctionality (**Figure 6**). Specifically, longitude (standardized path coefficient [SPC]=0.22, *P<*0 .05), mean annual precipitation (SPC=0.87, *P* < 0.01), climate seasonality (SPC=0.90, *P*< 0.01), and SD-beta (SPC=0.17, *P* < 0.05) were positively correlated with ecosystem multifunctionality. In addition to the direct effect, longitude indirectly affected ecosystem multifunctionality by affecting resource conditions, SD-beta, and climate seasonality (**Figure 6**). Similarly, maximum height was negatively correlated with longitude (SPC=-0.14, *P* < 0.01), but positively correlated with resource conditions (SPC=0.49, *P*<0.001) and climate stability (SPC=0.35, *P*< 0.01), which mainly resulted from the relationships between maximum height and mean annual precipitation and precipitation stability (**Figures 4D, E**). Both resource conditions and climate stability were driven by longitude with the standardized path coefficient of 0.63 and -0.65, respectively (**Figure 6**). Although FD-beta and PD-beta were affected by environmental factors, their effects on ecosystem stability were not significant (**Figure 6**; *P*> 0.05).

## 4 Discussion

### 4.1 Ecosystem multifunctionality and maximum height affected by precipitation gradients

In this study, we conducted field transect observations to study changes in multifunctionality and maximum height of shrub communities. We found that, with increasing longitude, ecosystem multifunctionality increased significantly (**Figure 5A**). This is similar to the results of previous research conducted in the arid regions of Northwest China (Zhang S. et al., 2022; Guo et al., 2023). We also found that the maximum height decreased with increasing longitude (**Figure 5B**). It is well known that with decreasing water and heat (increasing latitude), the aboveground wood yield and seed production all decrease, which is the result of many biological and abiotic factors (Moles et al., 2009; Gillman et al., 2015). Screening out the main ecological driving factors or potential biological-abiotic interaction processes is effective in explaining the causes of geographical patterns. In this study, the variations of ecosystem multifunctionality and maximum height with longitude were consistent with precipitation patterns (**Figures 1**, **2C, D**). These results indicated that precipitation is the most important ecological factor affecting ecosystem multifunctionality and maximum height of shrub communities.

### 4.2 Relationship between biodiversity and ecosystem multifunctionality is positive but weak

This study proves that species beta diversity, which increases with longitude, positively correlates with ecosystem multifunctionality (**Figures 5**, **6**). This result is similar to that of some studies showing that species beta diversity in arid regions may explain ecosystem multifunctionality better than species alpha diversity (Grman et al., 2018; Guo et al., 2023). In natural ecosystems, biodiversity can enhance ecosystem multifunctionality through resource (niche) complementarity among species (Soliveres et al., 2014). Therefore, the establishment of stable communities requires the maintenance of multiple functions by constructing different combinations of species in heterogeneous environments (i.e., beta diversity) (Grman et al., 2018; Guo et al., 2023). In other words, a community with a higher species pool should also have higher beta diversity, and thus, more reasonable cooperation between plants and resources (Thompson et al., 2018). Therefore, it is not surprising that species beta diversity, given resource constraints, exhibited stronger associations with ecosystem multifunctionality than species alpha diversity (**Figure 2E**). In addition, functional beta diversity strongly depends on the dominant species’ identity and traits (Gherardi and Sala, 2015). For instance, as the precipitation increases, the dominant species recur in specific plant types, with relatively large individuals exhibiting extensive resource utilization, which can contribute significantly to biomass (selection effect) (Grime, 1998). However, our sampling showed that the recurrence of dominant species was relatively low in the observed shrub communities (**Table S1**). Thus, in water-constrained arid regions, species can maximize multiple ecosystem functions by adjusting their ecological niches for rapid and massive reproduction (Soliveres et al., 2014; Yan et al., 2020). A previous study have shown that, under prolonged environmental stress, different species may evolve similar drought-tolerance traits, leading to a certain degree of functional redundancy (Hu et al., 2021). This corollary explains why functional and phylogenetic beta diversity are uncoupled from ecosystem multifunctionality (**Figure 6**).

The linkage between biodiversity and maximum height was proposed based on tree life form (Givnish et al., 2014; Stahl et al., 2014; Marks et al., 2016). However, whether this linkage exists in shrubs is unclear. In this study, we did not find biodiversity-driven relationships for maximum shrub height, which may be due to the narrow environmental gradients decreased species alpha diversity. In general, harsher environments (with narrow niche regions) will limit the variation of plant functional trait to a narrower spatial range, thus these regions should equilibrate at lower tree diversity than those with greater niche space (Tilman, 2004; Liang et al., 2022). A narrow ecological niche minimizes the difference in resources use between species, and different species (or vegetation patches) tend to develop similar resistance traits, such as lower specific leaf area and wood density, but higher water use efficiency to maintain survival (Cao et al., 2020; Page et al., 2011). Under the condition of water shortage in dryland, the investment of shrubs in the dimension of tree height does not have high returns compared with trees (McCulloh et al., 2015; Scheffer et al., 2014). Furthermore, the trade-off between rapid tree height growth rate to enhance competitiveness in benign environments and deep-rooted traits to maintain survival in harsh environments could also explain why many environmental tolerance species are rare or absent in more benign habitats, thus promoting beta diversity (Savage et al., 2013). However, this study did not find the relationship between beta diversity and maximum shrub height, perhaps because the above ground height was not the primary measure determining the resilience of shrub communities (Scheffer et al., 2014). Considering other integrated traits, such as rooting depth, may be more effective measures of harsh environment associated with biodiversity.

### 4.3 Precipitation fluctuations in determining ecosystem multifunctionality and maximum height

In this study, we considered the deeper relationships between water, heat, and geographic patterns of ecosystem multifunctionality. As expected, we found that precipitation was the most important environmental factor driving the geographical patterns of ecosystem multifunctionality in shrub communities. Specifically, the increase in mean annual precipitation along the longitudinal gradient significantly promoted ecosystem multifunctionality (**Figure 4D**). Some studies have also confirmed that with an increase in precipitation, species abundance and richness are both positively correlated with ecosystem multifunctionality in dryland ecosystems (Hu et al., 2021; Zhang S. et al., 2022; Guo et al., 2023). That is, in regions with less precipitation, lower interspecific competition may decrease the ability of species alpha diversity to maintain ecosystem multifunctionality (Hu et al., 2022). Furthermore, mean annual precipitation can be used to select niche-like plant species. For example, Saiz et al. (2019) and Berdugo et al. (2019) showed that decreased mean annual precipitation and increased precipitation seasonality could limit the migration of dominant species, thereby increasing the heterogeneity of plant communities in drylands. Under these conditions, the increase in the spatial heterogeneity of species distribution driven by precipitation may be the main reason for the correlation between ecosystem multifunctionality and species beta diversity.

The negative correlation between maximum height and longitude may be attributed to a decrease in precipitation stability along the longitude (**Figure 5B**). Many studies have shown that precipitation seasonality in Northwest China gradually increases from west to east (Zhang et al., 2018; Chen et al., 2023), which means that with an increase in precipitation, extreme rainfall events lead to a rapid reduction in water availability to vegetation in drylands (Zhang et al., 2018; Chen et al., 2023). A more stable and effective precipitation recharge is crucial for maintaining vegetation growth in dryland ecosystems. Our study demonstrates that the linkages between biodiversity and ecosystem multifunctionality vary along longitudinal gradients driven by precipitation regimes. At the same time, our findings highlight the importance of interpreting the biodiversity-maximum height relationships in conjunction with individual life form to capture the potential mechanisms in ecologically vulnerable regions.

## 5 Conclusion

Based on an extensive dataset of shrub communities in Northwest China, this study presents evidence that the longitudinal patterns of the ecosystem multifunctionality and maximum height in shrub communities are driven by precipitation. There was a significant positive relationship between species beta diversity and ecosystem multifunctionality at the regional spatial scale in the observed natural shrub communities. High precipitation seasonality and stability are important environmental factors that promote ecosystem multifunctionality and maximum height, respectively, in shrub communities. Overall, the regulation effects of precipitation fluctuation on ecosystem multifunctionality, maximum height, and biodiversity provide new insights into understanding the effects of global changes on ecosystem functioning.

## Data availability statement

The raw data supporting the conclusions of this article will be made available by the authors, without undue reservation.

## Author contributions

LD: Writing – review & editing. ST: Investigation, Data curation, Writing – review & editing. JS: Investigation, Data curation, Writing – review & editing. BZ: Investigation, Data curation, Writing – review & editing. XM: Investigation, Data curation, Formal Analysis, Writing – review & editing. LT: Data curation, Funding acquisition, Methodology, Writing – review & editing. XZ: Funding acquisition, Methodology, Writing – review & editing. YL: Funding acquisition, Methodology, Writing – review & editing.
