# Supplementary material for: Ecosystem multifunctionality, maximum height, and biodiversity of shrub communities affected by precipitation fluctuations in Northwest China
Source: Front Plant Sci. 2023 Sep 25;14:1259858. doi: 10.3389/fpls.2023.1259858 (PMC10560859; doi:10.3389/fpls.2023.1259858)
Supplement: Supplementary file 1 [file DataSheet_1.pdf]

Ecosystem multifunctionality, maximum height, and biodiversity of shrub communities affected by precipitation fluctuations in Northwest China

Supplementary Material

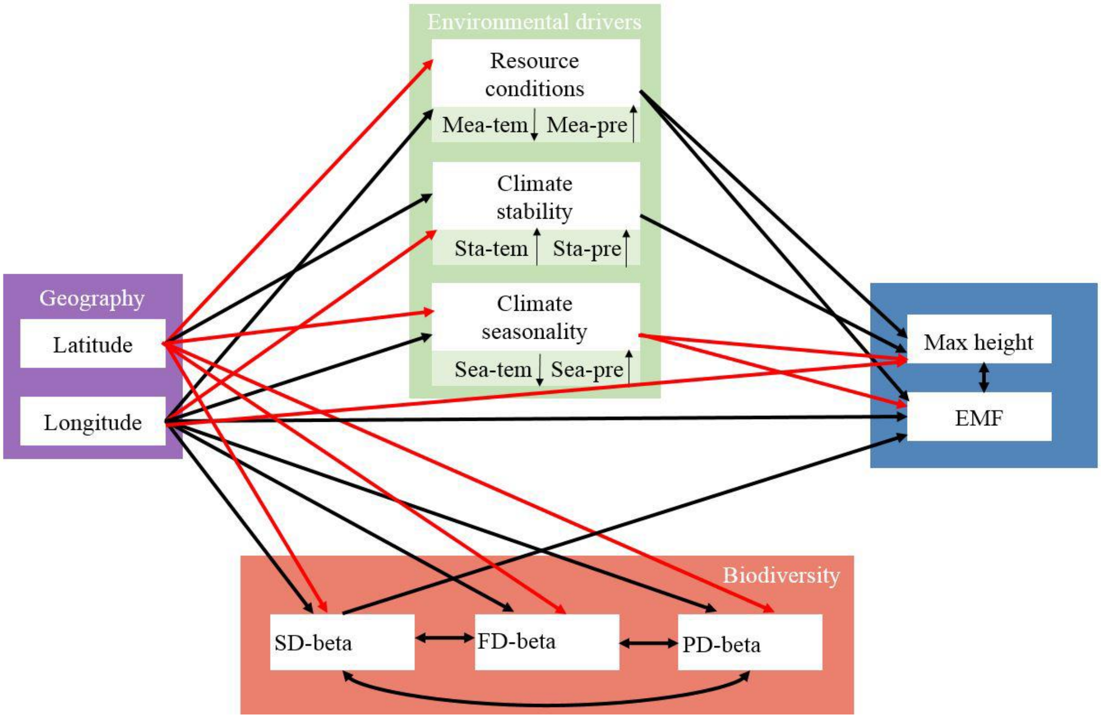

Figure S1 The initial pSEM framework based on priori knowledge. Single-headed arrows and double-headed arrows represent causal pathways and co-varying relationships, respectively. Black and red arrows represent positive and negative relationships, respectively. '↑' and '↓' represent the positive and negative relationships between variables and the corresponding PC1, respectively. Mea-pre, mean annual precipitation; Mea-tem, mean annual temperature; Sta-pre, precipitation stability; Sta-tem, temperature stability; Sea-pre, precipitation seasonality; Sea-tem, temperature seasonality; SD-beta, species beta diversity; FD-beta, functional beta diversity; PD-beta, phylogenetic beta diversity; EMF, ecosystem multifunctionality; Max height, mximum height.

Table S1 Summary of the natural assembly of shrub plants communities in Northwest China. Constructive species at each site was indicated in bold font.

Site ID	Site name	Latitude	Longitude	Elevation (m)	Dominant species
A	East of Karamy	45.35556	85.00472	280	*Nitraria tangutorum; Tamarix ramosissima; Anabasis brevifolia;* ***Haloxylon ammodendron***
B	Karamay Xiaoguai Township	45.175	84.98278	290	*Nitraria tangutorum; Tamarix ramosissima; Reaumuria songarica;* ***Haloxylon ammodendron***
C	Baijiantan, Karamy	45.49722	85.50667	260	*Nitraria tangutorum; Tamarix ramosissima; Anabasis brevifolia; Reaumuria songarica;* ***Haloxylon ammodendron***
D	Baijiantan, Karamy	45.50556	85.2075	280	*Anabasis brevifolia;* ***Haloxylon ammodendron****; Kalidium foliatum*
E	Mosowan Station	45.12278	86.02806	340	***Tamarix ramosissima****; Reaumuria songarica; Nitraria tangutorum; Haloxylon ammodendron*

F	Wucaiwan	44.16333	88.70889	610	***Tamarix ramosissima****; Anabasis brevifolia; Reaumuria songarica; Haloxylon ammodendron; Suaeda microphylla; Kalidium foliatum*
G	Wucaiwan	44.76167	89.21806	540	*Nitraria tangutorum; Anabasis brevifolia; Ephedra major; Reaumuria songarica; Calligonum mongolicum;* ***Haloxylon ammodendron***
H	Qitai	44.85222	90.01833	650	***Haloxylon ammodendron****; Halostachys caspica*
I	Mulei mingsha mountain	44.39583	90.64639	830	*Anabasis brevifolia; Reaumuria songarica;* ***Haloxylon ammodendron****; Kalidium foliatum*
J	Mulei	44.07444	90.44778	920	***Nitraria tangutorum****; Krascheninnikovia ceratoides*
K	Qitai Zhisha station	44.20028	90.14361	760	***Haloxylon persicum****; Atraphaxis bracteata; Anabasis brevifolia; Calligonum mongolicum; Artemisia ordosica; Haloxylon ammodendron; Krascheninnikovia ceratoides*
L	Hongliugou, Altay	45.28722	90.165	1200	*Anabasis brevifolia; Reaumuria songarica;* ***Haloxylon ammodendron***

M	Dure town	46.93167	88.90306	830	*Anabasis brevifolia;* ***Haloxylon ammodendron***
N	Fuhai Urungu River	46.72444	87.72556	570	*Salsola laricifolia; Tamarix ramosissima; Caragana halodendron; Reaumuria songarica;* ***Haloxylon ammodendron****; Krascheninnikovia ceratoides*
O	Valley zone	45.98111	85.84111	280	***Tamarix ramosissima****; Anabasis brevifolia Reaumuria songarica; Haloxylon ammodendron*
P		46.01194	86.41222	320	***Tamarix ramosissima***
					Nitraria roborowskii
					Nitraria sibirica
					Haloxylon ammodendron
					Kalidium foliatum
					Kalidium foliatum
Q	Mazong Mountain	41.81778	97.03611	1740	*Nitraria tangutorum; Reaumuria songarica;* ***Haloxylon ammodendron****; Halostachys caspica*
R	Ejin Banner	42.20583	101.1	920	***Tamarix ramosissima***
S	Ejin Banner	41.8675	100.5478	970	*Nitraria tangutorum;* ***Tamarix ramosissima****; Lycium ruthenicum*

T	Ejin Banner	41.81556	100.4681	980	*Nitraria tangutorum;* ***Tamarix ramosissima****; Reaumuria songarica; Calligonum mongolicum*
U		41.69694	103.1325	1010	***Reaumuria songarica***
V	Su Hongtu	41.29944	104.1436	800	*Nitraria tangutorum;* ***Tamarix ramosissima****; Haloxylon ammodendron; Kalidium foliatum*
W	Urigi	40.73306	104.51	1320	***Nitraria tangutorum****; Reaumuria songarica*
X	Taiyangmiao plantation	40.85806	106.7419	1040	***Haloxylon ammodendron***
Y	Narin lake	40.56528	106.3758	1040	*Nitraria tangutorum; Tamarix ramosissima;* ***Haloxylon ammodendron***
Z	Narin lake	40.57333	106.3667	1050	*Nitraria tangutorum;* ***Sarcozygium xanthoxylum****; Ammopiptanthus mongolicus*
A1		40.76139	105.3769	1180	*Nitraria tangutorum; Potaninia mongolica; Reaumuria songarica;* ***Kalidium foliatum***
B1	Yellow River Bridge	40.66389	107.5636	1040	*Artemisia ordosica;* ***Salix cheilophila***
C1	Erdos	40.43	108.6567	1120	*Artemisia ordosica;* ***Salix cheilophila***

D1		40.36333	109.4231	1080	***Corethrodendron scoparium****; Caragana sinica; Artemisia ordosica*
E1	Engebesa Lake	40.37222	109.4431	1070	*Caragana sinica; Artemisia ordosica;* ***Salix cheilophila***
F1		40.20556	110.8397	1040	*Caragana sinica; Artemisia ordosica;* ***Salix cheilophila***
G1		39.36333	111.1156	890	***Caragana sinica****; Artemisia ordosica*
H1	Erdos	39.71639	105.9086	1170	***Caragana sinica****; Artemisia ordosica*
I1		39.68917	110.1378	1490	*Caragana sinica;* ***Hippophae rhamnoides****; Kalidium foliatum*
J1		39.77139	109.3739	1370	*Caragana sinica; Artemisia ordosica;* ***Salix cheilophila***
K1		39.84111	108.6264	1370	*Atraphaxis bracteata;* ***Corethrodendron scoparium****; Caragana sinica; Artemisia ordosica*
L1		40.12694	107.6139	1200	***Caragana sinica****; Kraschenninnikovia ceratoides*

M1	Balagon	40.25639	107.0706	1140	*Nitraria tangutorum; Sarcozygium xanthoxylum;* ***Caragana sinica****; Ammopiptanthus mongolicus; Artemisia ordosica; Tetraena mongolica; Reaumuria songarica; Juniperus sabina; Reaumuria songarica*
N1		39.87389	106.8825	1230	*Sarcozygium xanthoxylum; Ammopiptanthus mongolicus; Artemisia ordosica; Tetraena mongolica; Potaninia mongolica;* ***Juniperus sabina***
O1	Northwest of Mu Us Desert	38.98222	107.3258	1180	*Nitraria tangutorum; Reaumuria songarica;* ***Ammopiptanthus mongolicus****; Kalidium foliatum; Reaumuria songarica*
P1		38.71306	106.8856	1240	***Artemisia ordosica***
Q1		37.72694	107.4972	1330	***Caragana sinica***
R1		37.81778	108.0008	1360	*Corethrodendron scoparium; Caragana sinica; Artemisia ordosica;* ***Salix cheilophila****; Amorpha fruticosa*
S1		37.85333	108.3736	1310	*Caragana sinica; Artemisia ordosica;* ***Salix cheilophila***
T1		38.46722	108.7706	1360	***Caragana sinica****; Artemisia ordosica*

U1	38.97028	109.3197	1310	***Juniperus sabina**; Caragana sinica; Artemisia ordosica; Salix cheilophila*
V1	39.27556	108.9506	1310	*Corethrodendron scoparium; Caragana sinica; Artemisia ordosica; Hippophae rhamnoides;* ***Salix cheilophila***
W1	39.01778	108.0258	1370	***Artemisia ordosica***
X1	39.4575	106.6436	1230	*Nitraria tangutorum; Sarcozygium xanthoxylum ;* ***Caragana sinica****; Potaninia mongolica; Reaumuria songarica; Ammopiptanthus mongolicus; Tetraena mongolica; Krascheninnikovia ceratoides*
Y1	39.1075	105.7017	1660	***Corethrodendron scoparium****; Calligonum mongolicum; Artemisia ordosica*
Z1	37.97639	105.3394	1330	***Nitraria tangutorum****; Reaumuria songarica*
A2	37.43861	104.3678	1640	*Nitraria tangutorum;* ***Reaumuria songarica****; Caroxylon passerinum*
B2	37.57472	103.7125	1750	***Nitraria tangutorum****; Caragana sinica; Artemisia ordosica*
C2	37.72972	103.1478	1660	***Corethrodendron scoparium****; Artemisia ordosica*

D2	38.31194	103.26	1470	***Nitraria tangutorum****; Reaumuria songarica*
E2	38.27861	103.9267	1400	***Nitraria tangutorum***
F2	38.30167	104.4778	1360	***Nitraria tangutorum****; Kalidium foliatum*
G2	38.72	105.3047	1280	***Corethrodendron scoparium****; Artemisia ordosica*
H2	39.55083	105.5978	1050	***Nitraria tangutorum****; Ammopiptanthus mongolicus; Calligonum mongolicum; Artemisia ordosica*
I2	39.68528	105.0811	1320	*Nitraria tangutorum; Reaumuria songarica; Kalidium foliatum;* ***Salsola laricifolia****; Caroxylon passerinum*
J2	40.215	104.1806	1440	***Nitraria tangutorum****; Reaumuria songarica*
K2	39.73361	103.2603	1280	*Nitraria tangutorum;* ***Artemisia ordosica***
L2	39.41556	102.4797	1340	*Nitraria tangutorum;* ***Sarcozygium xanthoxylum****; Artemisia ordosica*
M2	39.19417	101.5108	1420	*Nitraria tangutorum; Reaumuria songarica;* ***Kalidium foliatum****; Caroxylon passerinum*

N2		39.58861	100.7969	1510	*Nitraria tangutorum; Reaumuria songarica;* ***Kalidium foliatum****; Caroxylon passerinum*
O2		40.13861	100.1114	1240	***Nitraria tangutorum****; Reaumuria songarica; Calligonum mongolicum*
P2		39.865	98.64944	1400	*Nitraria tangutorum;* ***Reaumuria songarica****; Calligonum mongolicum*
Q2		40.71806	96.67833	1510	***Reaumuria songarica***
R2		42.14528	95.8775	1820	*Reaumuria songarica;* ***Haloxylon ammodendron***
S2		43.12028	95.30444	1130	***Ephedra major***
T2		44.21472	87.86083	490	*Sarcozygium xanthoxylum;* ***Tamarix ramosissima****; Reaumuria songarica; Kalidium foliatum*
U2	Cainan highway	44.345	88.135		***Tamarix ramosissima****; Anabasis brevifolia; Reaumuria songarica; Haloxylon ammodendron*
V2	Southern edge of Gurbantunggut Desert	44.73333	88.45	465.5	*Nitraria roborowskii;* ***Tamarix ramosissima****; Reaumuria songarica; Kalidium foliatum; Caragana halodendron; Nitraria sibirica*

W2	Southern edge of Gurbantunggut Desert	44.39778	87.92611	450.8	*Haloxylon persicum; Tamarix ramosissima;* ***Haloxylon ammodendron***
X2	Southern edge of Gurbantunggut Desert	44.37917	87.94389	470	***Tamarix ramosissima****; Reaumuria songarica; Haloxylon ammodendron*
Y2	Southern edge of Gurbantunggut Desert	44.41556	87.91611	453.95	***Haloxylon persicum****; Reaumuria songarica; Haloxylon ammodendron*
Z2	Southern edge of Gurbantunggut Desert	44.37278	87.9375	460	*Haloxylon persicum;* ***Haloxylon ammodendron***
